# Influence of Preparation Methods and Nanomaterials on Hydrophobicity and Anti-Icing Performance of Nanoparticle/Epoxy Coatings

**DOI:** 10.3390/polym16030364

**Published:** 2024-01-29

**Authors:** Shinan Liu, Houzhi Wang, Jun Yang

**Affiliations:** 1 School of Transportation, Southeast University, Nanjing 211189, China; 230238926@seu.edu.cn (S.L.); yangjun@seu.edu.cn (J.Y.); 2 Key Laboratory of Transport Industry of Comprehensive Transportation Theory (Nanjing Modern Multimodal Transportation Laboratory), Ministry of Transport, Nanjing 211135, China

**Keywords:** hydrophobic coating, nanoparticles, ice adhesion force, contact angle, asphalt concrete

## Abstract

Despite their effectiveness in preventing icing, hydrophobic coatings possess drawbacks such as susceptibility to detachment and limited wear resistance, leading to inadequate longevity in melting ice/snow. To enhance the surface stability and durability of superhydrophobic coatings, nanoparticle/epoxy formulations were developed using three types of nanoparticles, two dispersion techniques, three application methods, and two epoxy resin introduction approaches. Testing encompassed water contact angle measurements, assessment of ice adhesion force, and determination of icing rates on asphalt concrete coated with these hydrophobic formulations. Fourier-transform infrared spectroscopy was employed to analyze the molecular structures of the coatings, while scanning electron microscopy facilitated observation of the surface morphology of the hydrophobic coatings. The findings indicated that nano-ZnO, TiO_2_, and SiO_2_ particles could be modified into hydrophobic forms using stearic acid. Application of the hydrophobic coating improved the concrete’s hydrophobicity, reduced ice adhesion strength on both concrete and asphalt, and delayed the onset of icing. Furthermore, optimal dosages of stearic acid, nanoparticles, and epoxy resin were identified as crucial parameters within specific ranges to ensure the optimal hydrophobicity and durability of the coatings.

## 1. Introduction

In winter, most expressways face the problem of road icing, which significantly reduces the friction coefficient of the road surface and leads to serious traffic accidents. In addition, the resulting freeze–thaw damage may shorten the road lifespan. Moreover, because the surface of the concrete is porous, water enters the surface. In particular, the water in the pores freezes and expands, resulting in tensile stress inside the concrete and cracking of the concrete. Furthermore, the ice in the pores is integrated with the surface ice; consequently, the ice is firmly attached to the concrete surface, which hampers deicing.

For the problem that concrete pavement is easy to freeze and difficult to remove ice from, a great deal of research has been conducted on the icing behavior of concrete road surfaces, and many active anti-icing and passive deicing technologies for concrete pavement have been applied [1,2,3,4,5,6,7]. The active anti-icing method consists of modifying the traditional material and structural design of the pavement before icing occurs. This approach mainly yields self-stress elastic pavement, low-freezing-point pavement, and energy conversion pavement. However, these technologies are not widely used owing to their capital cost and construction difficulty. The passive deicing method consists of removing the ice by physical and chemical means when icing is complete. This approach is mainly categorized into mechanical deicing and chemical agent deicing. Research and engineering practice have proved that passive deicing not only damages the road surface seriously but also has short-term effects.

Removing water from the concrete prevents the freezing of the concrete surface totally. Superhydrophobic coatings (advancing contact angle larger than ~150° and contact angle hysteresis of less than ~10°, or roll-off angle of less than ~5°) [8] are waterproof, antifog, and anti-icing and are widely used in aerospace, power communication, etc. [9,10,11,12,13]. However, few hydrophobic coating technologies are applied to road surfaces. In 1997, German botanist Barthlott [14] found that the surface of lotus leaves has strong hydrophobicity and self-cleaning ability; this captured the attention of scholars worldwide, who carried out in-depth research on hydrophobic materials. In 2002, Laforte [15] first discovered that superhydrophobic coatings can reduce the ice adhesion to the substrate, and the hydrophobic anti-icing technology has developed rapidly since then. Arabzadeh et al. [16] mixed polytetrafluoroethylene, epoxy resin, and acetone to obtain a superhydrophobic coating for pavements, which has a good anti-icing effect. Ruan [17] prepared an anti-icing hydrophobic coating on the surface of an aluminum alloy substrate. The produced hydrophobic surface did not start to freeze until the temperature reached −8 °C, indicating that it can reduce the freezing temperature. Yang [18] prepared a hydrophobic coating on an aluminum surface and achieved significant anti-icing properties; in particular, the surface icing was greatly delayed. Antonini [19] used an aluminum substrate to study the effect of hydrophobic coatings on the anti-icing of aircraft wings. The results showed that a hydrophobic coating can reduce not only the wing icing but also the energy used to deice the wing (by 80%). Dotan [20] studied the relationship between water wettability and ice adhesion. Various polycarbonate-coated surfaces were obtained through appropriate chemical methods, including superhydrophilic and superhydrophobic surfaces. Ice adhesion tests and contact angle measurements showed that the larger the contact angle, the lower the ice adhesion. The best results were obtained in the case of superhydrophobic surface treatments, where ice adhesion was significantly reduced compared to untreated aluminum surfaces. However, the main focus of current research on superhydrophobic nanocoatings is metal anticorrosive rather than road anti-icing coatings [21,22]. On account of the different characteristics of asphalt concrete, cement concrete, and metal substrates, road anti-icing coatings require further research.

The addition of polar nanoparticles can reduce the surface energy and enhance the hydrophobicity of the coating [23]. However, the high surface energy of nanoparticles prevents them from dispersing uniformly in organic media, which limits the application of nanocoatings [24,25]. To solve this problem, researchers found that the organic modification of nanoparticle surface can achieve a better dispersion effect [26,27,28,29].

Existing studies show that improving the hydrophobicity of the coating and the adhesion between the coating and the road surface can achieve good deicing performance and durability [30,31]. However, the current research on the application of hydrophobic materials for road anti-ice condensation is mostly limited to laboratory experiments, and few coating technologies are used in physical engineering and are recognized and promoted. This is because the superhydrophobic coating material is brittle and susceptible to wear, has weak adhesion to the road surface, falls off easily, and has poor surface stability, which leads to insufficient durability of the ice and snow melting function [32,33]. Therefore, to solve the defects in coating technology, epoxy and nanomaterial coatings were designed and prepared in this study not only to realize the anti-icing and deicing of asphalt pavement but also to improve the wear, water, and impact resistance of asphalt pavement coating. Further, there are few evaluation methods for the anti-icing performance of coatings, and few repeatable and standardized test methods that can quantitatively analyze the anti-icing performance of coatings or asphalt pavements [34,35,36]. Through evaluation tests of anti-icing performance, a quantitative and reproducible evaluation method for the anti-icing performance of coatings and a test platform were developed in this study. Based on the proposed method, the anti-icing performance of the hydrophobic epoxy coating was tested at low temperatures and the anti-icing performance mechanism of coatings was explored from the perspective of microstructure.

In this study, the research aimed to assess the potential of nanoparticles as coating materials for asphalt mixtures and their impact on enhancing pavement coating durability by incorporating epoxy resins. The investigation systematically analyzed the influence of preparation methods and nanomaterials on the hydrophobicity and ice resistance of nanoparticle/epoxy coatings. Initially, formulations combining three nanoparticle types, two dispersion techniques, three application methods, and two epoxy resin introduction approaches were developed to determine the optimal coating preparation method. Subsequently, assessments of hydrophobic and anti-icing properties were conducted through contact angle tests and adhesion strength assessments. Finally, the study delved into understanding the variations in hydrophobicity among nanoparticles and investigated the mechanism underlying the enhanced durability afforded by epoxy resin, utilizing Fourier-transform infrared (FT-IR) spectroscopy and scanning electron microscopy (SEM) analysis.

## 2. Materials and Methods

### 2.1. Raw Materials

ZnO, TiO_2_, and SiO_2_ nanoparticles were used as the main component for hydrophobic coatings, produced by Zhejiang Yuante New Material Co., Ltd. (Huzhou, China). Their average particle sizes were 17, 15, and 30 nm, respectively. All the reagents used in the experiment were analytically pure, including stearic acid, produced by Fuchen Chemical Reagent Co., Ltd. (Tianjin, China); and anhydrous ethanol, produced by Shandong Usolf. Stearic acid proves to be a more cost-effective and environmentally conscious hydrophobic modifier compared to alternatives like fluorosilicone. The epoxy resin (bisphenol-A) utilized in this research was supplied by Baling Petrochemical, Sinopec Asset Management Co., Ltd. (Beijing, China). The curing agent was sourced from Dayou Construction Co., Ltd. (Tianjin, China). For simplicity, the material resulting from the curing process involving epoxy resin and the curing agent will henceforth be referred to as epoxy resin in the subsequent article. Epoxy resin was employed for two functions: (1) To improve the wear, water, and impact resistance of the coating, epoxy resin was mixed with hydrophobic materials as paint and diluent. (2) To improve the bonding between the coating and asphalt pavement, epoxy resin adhesive layer was employed between the hydrophobic coating slurry and the pavement surface. Stearic acid was used as a modifier for ZnO, TiO_2_, and SiO_2_ nanoparticles. The AC-16 grading was applied for the asphalt mixture in the preparation of Marshall specimen. A Pen70 asphalt was used in the preparation of Marshall specimen. The main basic properties of the Pen70 asphalt are listed in Table 1.

The AC-16 asphalt mixture was designed in accordance with T 0702–2011 technical specifications [37]. The content ratio of asphalt binder to aggregates was 4.6%. After heating the aggregates at 170 °C for 6 h, the heated aggregates were incorporated and agitated for 60 s. Then, the heated asphalt binder at 165 °C was added and agitated for 90 s. At last, the asphalt mixtures were compacted double sided, and the standard Marshall specimens were obtained. The diameter of the specimen was 101.6 mm and the height of the specimen was 63.5 mm.

### 2.2. Preparation of the Nanoparticle Hydrophobic Materials

The detailed preparation steps of the nanoparticle hydrophobic materials are as follows:

Different amounts of stearic acid granules were dissolved in 100 mL absolute ethanol to form a transparent stearic acid absolute ethanol solution. Afterward, different amounts of nanoparticles were added to the stearic acid absolute ethanol solution, which was stirred in a magnetic stirrer (MS) for 3 h until the nanoparticles were completely dispersed to form a milky white solution. Similarly, another solution was stirred using an ultrasonic dispersion (UD) instrument and the solution was completely dispersed after 10 min.

### 2.3. Preparation of the Hydrophobic Coatings

#### 2.3.1. Application Method

In this study, to determine the optimum preparation method for the hydrophobic coatings, spraying, dipping, and brushing methods were used to apply the hydrophobic materials.

**Figure 1:**
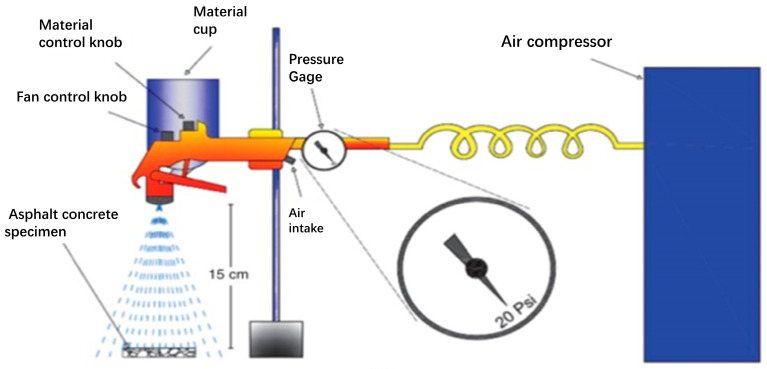
Diagram of spraying method.

Spraying method (SM): Dust and other pollutants were removed from the surface of the specimen with paper towel, which was dried thereupon. Spraying was carried out with a spray gun. The zinc oxide stearic acid solution was poured into the spray gun. The pressure of the spray gun was set to 20 Psi. The vertical distance of spraying was 15 cm away from the glass slide, and the spray gun was kept downward and vertical. The glass slide was sprayed continuously for 15 s (10 times) and thereafter kept horizontally at 25 °C for 24 h, as shown in Figure 1.

Dipping method (DM): Dust and other pollutants were removed from the surface of the specimen, which was dried thereupon. The clean specimen was immersed in the zinc oxide stearic acid solution for 30 s and thereafter kept horizontally at 25 °C for 24 h.

Brushing method (BM): Dust and other pollutants were removed from the surface of the specimen, which was dried thereupon. A certain amount of zinc oxide stearic acid solution was brushed on the specimen three times using a brush with a width of 5 cm 3 times to ensure that the coating was evenly distributed on the surface of the glass slide. Afterward, the glass slide was kept horizontally at 25 °C for 24 h.

#### 2.3.2. Preparation of Coated Marshall Specimens

Epoxy resin has excellent physical and mechanical properties, electrical insulation properties, and adhesion properties with various materials. Therefore, it is used in coatings, composites, castings, adhesives, molding materials, and injection molding materials. Epoxy resin confers good strength and durability on the coating, but it is easy to crack and has poor impact resistance owing to its high stiffness, especially in a low-temperature environment.

Therefore, a nanoparticle/epoxy composite coating was prepared in this study. The epoxy resin improved the coating durability, and the hydrophobic nanomaterials ensured the hydrophobicity and anti-icing properties of the coating, so that the coating could meet the road performance.

For the two functions of epoxy resin, two preparation methods of coated Marshall specimens were designed.

(1) Preparation of nanoparticle/epoxy hybrid coatings

First, 5 g epoxy resin was introduced into the hydrophobic materials and the mixed solution was dispersed using a magnetic stirrer for 10 min. Then, coating slurry was applied on the surface of Marshall specimen by the optimum preparation method. To determine the optimum mass ratios of nanoparticles to stearic acid, we set different mass ratios of each substance in the hydrophobic materials, as shown in Table 2. To research the effect of mass ratio of nanoparticle to stearic acid on contact angle, we set different mass ratios.

(2) Preparation of ZnO/epoxy layered coating

First, 5 g epoxy resin was applied on the surface of Marshall specimen, and then different amounts of hydrophobic material were applied after the epoxy resin began to cure and was not fully cured (2 days at 60 °C) with a certain viscosity. The mass ratios of epoxy resins to hydrophobic materials are listed in Table 2.

### 2.4. Characterization of Hydrophobic Materials and Coatings

In this study, to determine the optimum preparation method, the contact angles of hydrophobic coatings were measured. The volume of the liquid used for each measurement was 4 μL and three different positions were taken for the contact angle test. The sessile drop and tangent searching fitting modes were applied to fit the contact angle using the software of the contact angle detector [38].

Since the static contact angle does not contain any meaningful information about the wetting state of the surface [8], the wetting analysis was performed by measuring the advancing and receding contact angle (ACA and RCA) with careful protocol [8,39]. The measurement procedure for advancing and receding contact angle strictly follows the literature method [39]. For convenience, the arithmetic mean of ACA and RCA is used as an estimate of equilibrium contact angle (ECA) [40]. Contact-angle hysteresis (CAH) was defined as the difference between the ACA and the RCA [41]. Five different positions were taken for the contact angle test.

The microstructures of the hydrophobic materials were observed using field-emission scanning electron microscopy (FESEM, Nova Nano SEM 450) with an accuracy of up to 30 nm. Because the measurements required objects with high conductivity, a metal layer was placed on the hydrophobic coating before the observation.

The chemical structures of samples were characterized by FTIR spectroscopy (Nicolet iS10). The samples were prepared using the potassium bromide tablet method. The test ranges were from 500 cm^−1^ to 4000 cm^−1^. To get rid of impurities, the nanomaterials were centrifuged and cleaned with distilled water and alcohol before the FTIR test.

### 2.5. Adhesion Test

**Figure 2:**
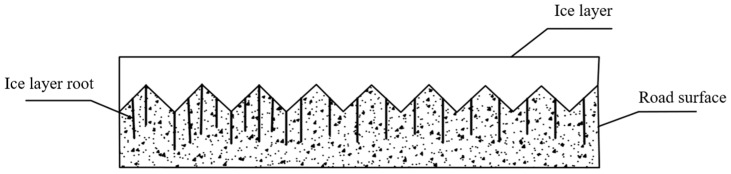
Interlocking of ice–pavement interface.

Due to the rough and porous nature of the pavement surface, a phenomenon arises at the ice–pavement interface involving interlocking and adhesion when icing exceeds 105 nm in thickness [30,42], as shown in Figure 2.

Reducing interlocking at the ice–pavement interface involves employing a combined vertical pulling force and horizontal shear force, which is a conventional road de-icing method. Adhesion strength testing methods commonly include the horizontal shear test and tensile test [34,35,36]. 

**Figure 3:**
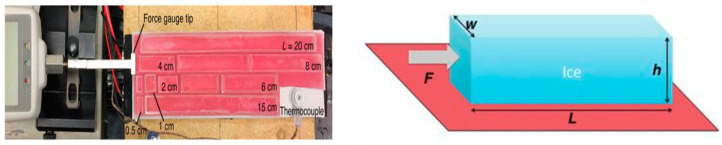
Method for evaluating the adhesion strength by GOLOVIN et al. [43], reproduced from [43], with permission from Science, 2023.

The efficacy of coatings has been evaluated by the adhesion force of the ice layer to the road surface and ice area to characterize the pavement force to remove ice layer from the iced pavement. GOLOVIN et al. evaluated the adhesion strength by calculating the force required to push the ice cube and the contact area of the ice cube with the base, as shown in Figure 3 [43].

**Figure 4:**
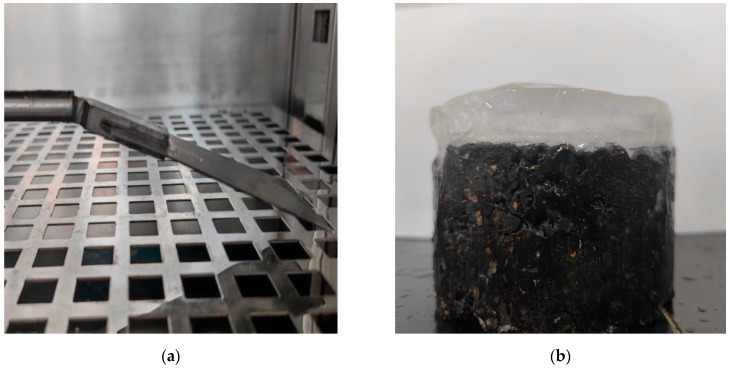

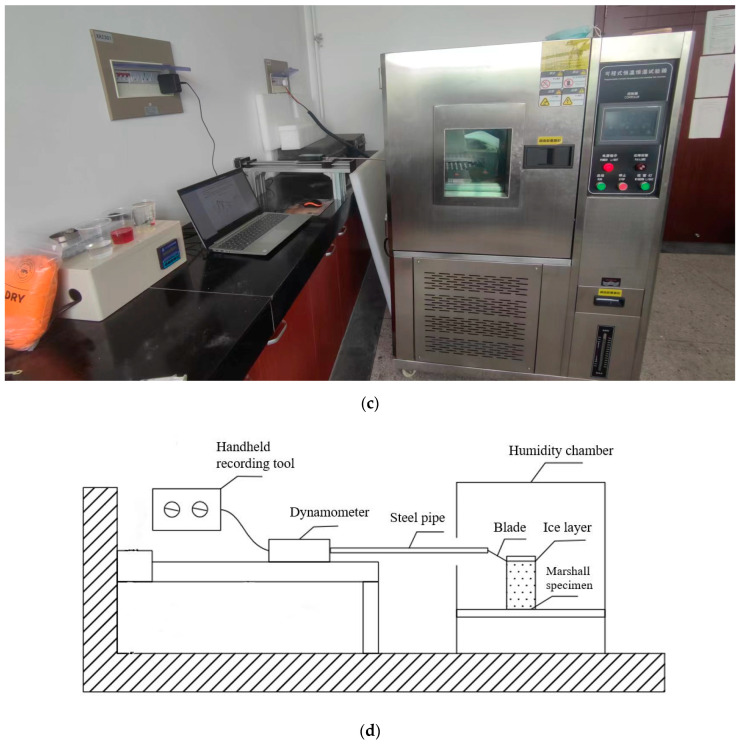
Evaluation method for adhesion force: (**a**) blade; (**b**) iced Marshall specimen; and (**c**) temperature humidity test chamber; (**d**) experiment instrument.

Based on the method of GOLOVIN et al., a reproducible and quantitative method was designed to test the ice adhesion force on the surface and realize the quantitative evaluation of the anti-icing performance of the pavement, as shown in Figure 4. To meet the requirements of the simulation of real-road deicing and quantitative data, a set of test equipment was designed to simulate manual deicing. The anti-icing performance of the road surface was characterized by the maximum force while shoveling the accreted ice in a specific area.

The design includes the following three points:

(1) Simulation of manual deicing

In the anti-icing evaluation test, the damage of the ice-covered layer consists of vertical pullout damage and horizontal shear damage. These two types, especially the vertical pullout damage, are quite different from those of manual deicing. The vertical pullout damage is caused by applying a vertical pulling force between the ice layer and the road surface. Since there is no horizontal component force, the force required to destroy the bond between the ice layer and the road surface is much larger than that required for deicing. A road can be deiced by exerting a lateral force on the ice-covered layer to the road surface at a certain angle (30°), and the resultant damage is the combined effect of vertical pulling force and horizontal shear force. However, GOLOVIN’s method only involves horizontal shear damage [43]. Therefore, to be practical, this study introduces an enhanced approach to assess coatings’ anti-icing efficacy by integrating horizontal and vertical force assessments based on GOLOVIN’s method. To be practical, this study customized a 50 × 50 mm^2^ blade, which was placed at an angle of 30° to the horizontal and was connected to a 750 mm cylindrical steel pipe; the pipe was connected to the controller to move horizontally. The simulation of manual deicing was controlled by the blade. In particular, when the blade removed the ice, the manual deicing was simulated.

(2) Measurement of surface ice adhesion

The other end of the steel pipe was connected to a dynamometer, which was placed on the mobile device of the controller, and deicing was dynamically measured in real time through the computer software handheld recording tool.

(3) Formation of ice-covered layer and control of the quantitative area

To simulate the freezing and low-temperature environment in winter, the test specimens (Marshall specimens) were first pre-frozen in a low-temperature environment for 15 h, simulating the winter road surface. To control the ice-covered layer to reach a quantitative area, the Marshall specimen was wrapped with tin foil to create a cylindrical water tank and sealed to prevent water from flowing out. Subsequently, the water was dripped on the surface of the test specimen until the thickness of the ice layer reached 2 cm. The specimens were frozen for 5 h at −10 °C until the ice layer was completely condensed on the surface.

The adhesion strength was characterized using Equation (1)

*τ_ice_* = *F*/*A* (1)

where *τ_ice_* is adhesion strength, (KPa); *F* represents the adhesion force, (kN); and *A* represents the area of ice (m^2^), *A* = πR^2^ = 3.14 × 0.10162/2 m^2^ = 0.00811 m^2^.

### 2.6. Simulation Test of Icing

As evaluation indexes of anti-icing performance, the amount and speed of road icing significantly influence the low-temperature safety and performance of the pavement. In the context of this investigation, we scrutinized the anti-icing capabilities of hydrophobic coatings applied to asphalt concrete by replicating the natural phenomenon of water accumulation on road surfaces, subsequently subjecting them to freezing conditions.

The beginning icing time of water was observed on the different hydrophobic surfaces of asphalt concrete to investigate the icing delay function of hydrophobic coatings. Several groups of coated and uncoated specimens were prepared and put into the constant temperature and humidity box at −10, −6, and −2 °C for 5 h. Then, 15 g of water was dripped on the surface of the Marshall specimen wrapped with tin foil. Subsequently, the unfrozen water was exported and weighed every 30 min to calculate the icing proportion. The icing proportion was calculated as the ratio of the mass of ice to the total mass of ice and water.

### 2.7. Durability Test of Coatings

Epoxy resin provides primary durability in coating. To study the effect of epoxy resin content on the durability of different coatings, the durability test was carried out on an experimental road with a traffic flow of 3000 pcu/d. Coating durability is characterized by the decay of the contact angle. Coatings with low attenuation of contact angle have better durability. The mass ratio of nanoparticles to stearic acid and the dosage of nanoparticle to hydrophobic materials were set to the optimum value. Coatings with different epoxy contents in sizes of 50 cm × 50 cm were applied on the asphalt pavement. At regular intervals of 20 days, we procured samples from these distinct coatings and meticulously gauged their contact angles. This multifaceted investigation sought to shed light on the intricate relationship between epoxy resin content and the ensuing durability of the coatings, within the context of real-world traffic conditions.

## 3. Results and Discussion

### 3.1. Determination of the Optimum Preparation Method

#### 3.1.1. Dispersion Method

To determine the optimum dispersion method, the contact angles of the ZnO nanoparticle hydrophobic materials coatings dispersed using the magnetic stirrer (MS) and ultrasonic dispersion (UD) instrument were measured. The spraying method was used for application.

**Figure 5:**
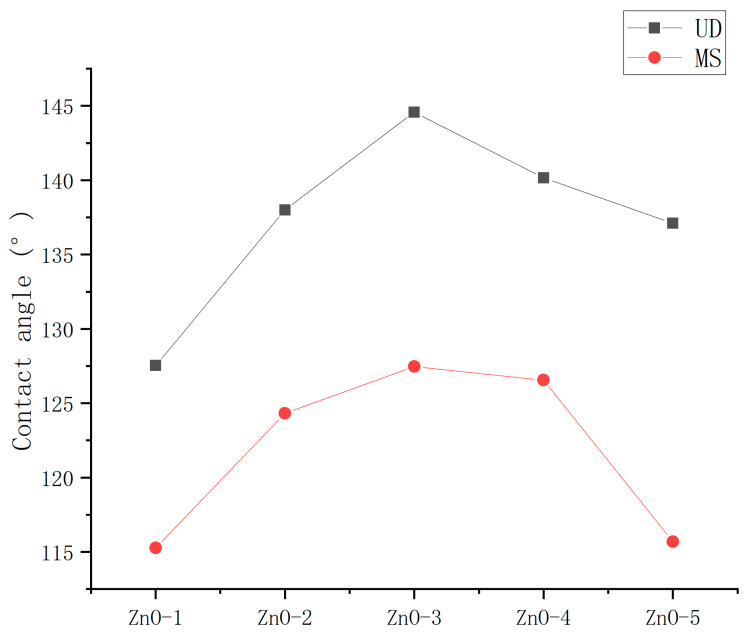
Contact angles of ZnO hydrophobic materials dispersed using MS and UD.

As shown in Figure 5, the contact angles of the coatings prepared by ultrasonic dispersion were less than magnetic stirring, which shows that both preparation methods had a great influence on the hydrophobicity of the coating. The optimum mass ratios of nanoparticles to stearic acid of the hydrophobic materials dispersed using MS and UD were the same, indicating that the dispersion method did not influence the optimum mass ratios of nanoparticles to stearic acid of the hydrophobic materials.

**Figure 6:**
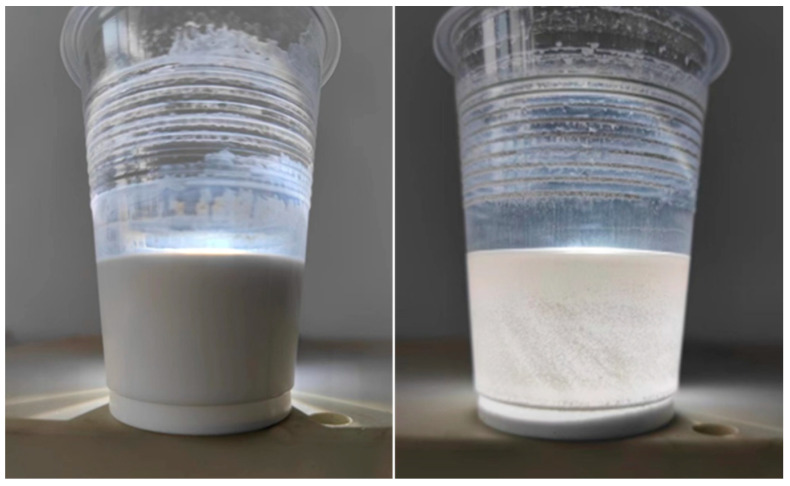
Ultrasonically dispersed (**left**) and magnetically stirred (**right**) ZnO-3 sample (after standing for 24 h).

As shown in Figure 6, the ultrasonically dispersed solution was homogeneous but the magnetically stirred solution had obvious stratification. There are three possible reasons: 1. The ultrasonic disperser can reduce the particle size. When the particles are small enough, they are suspended in the liquid owing to buoyancy, making the solution homogeneous, whereas the particle size of the material prepared by magnetic stirring is larger and precipitation occurs over time. 2. In contrast to magnetic stirring, the solution prepared by ultrasonic dispersion induces chemical reactions. 3. The combined effect of the first and second reasons.

To further explore the differences between the two solution dispersion methods, FTIR analysis of zinc oxide nanoparticles, magnetically stirred zinc oxide stearic acid particles, and ultrasonically dispersed zinc oxide stearic acid particles was carried out. The samples were prepared using the method in Section 2.2. The mass ratio of nanoparticle to stearic acid was 2.86:1.

**Figure 7:**
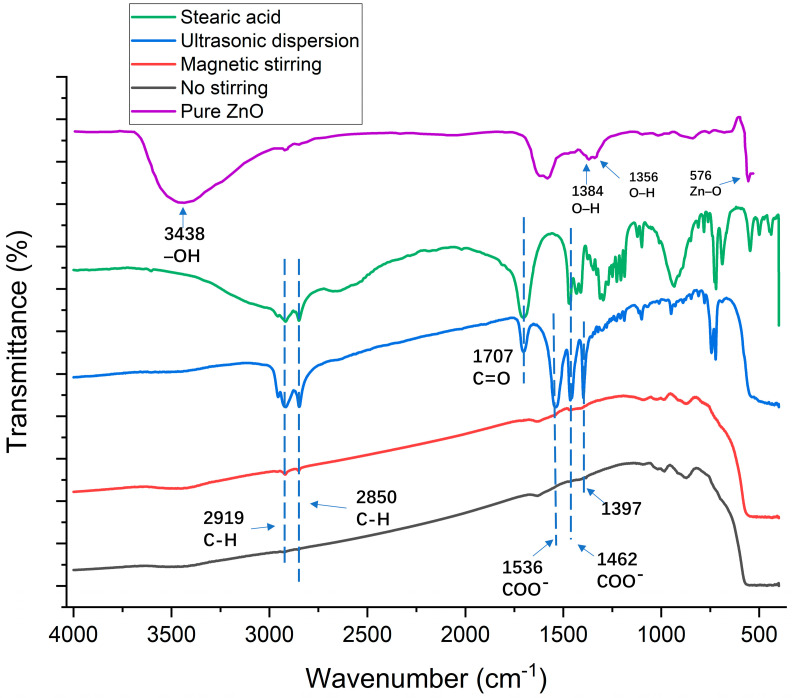
Fourier-transform infrared (FTIR) spectra for zinc oxide particles and modified zinc oxide particles using different preparation methods for coating solution.

As depicted in Figure 7, in pure ZnO, the 576 cm^−1^ peak was attributed to the stretching vibration absorption of Zn-O. Peaks at 1356 cm^−1^ and 1384 cm^−1^ were associated with primary and secondary alcohol in-plane bending or vibration. The stretching and absorption vibration peaks of hydroxyl groups on the ZnO surface were observed at 3438 cm^−1^. The unmodified nanomaterials exhibit a hydrophilic nature due to the substantial formation of hydroxyl groups on the nanoparticle surface via adsorption in conjunction with water molecules, influenced by the surface effect.

For modified ZnO, the hydroxyl groups at 3438 cm^−1^ disappeared. In addition, the peaks at 2919 and 2850 cm^−1^ were assigned to the asymmetric stretching vibration absorption of the C–H of the alkyl group in stearic acid. Stearic acid and all modified ZnO exhibit peaks at 2919 and 2850 cm^−1^ indicating that the alkyl group in stearic acid was grafted onto the surface of ZnO particles [44]. In addition, the area of the C–H peak of the ultrasonically dispersed, magnetically stirred, and unprocessed solutions showed a decreasing trend, indicating that the reaction degree of the three kinds of samples gradually decreased. The peak at 1707 cm^−1^ was assigned to the stretching vibration absorption of C=O. Since the absorption peak of the C=O functional group is a typical absorption peak in the molecular structure of stearic acid [45], the existence of the C=O functional group in the FTIR spectra of modified zinc oxide indicates that the reaction was not complete and an excess of stearic acid is still present. The peaks at 1536 and 1462 cm^−1^ were assigned to the asymmetric and symmetric, respectively, stretching vibration of the COO^–^ functional group, indicating that the hydroxyl group on the surface of the nanoparticles was replaced by the carboxyl group of stearic acid and existed on the surface of the nanoparticles in the form of coordination adsorption [46,47]. In contrast, these characteristic peaks did not appear in the FTIR spectra of the magnetically stirred and unprocessed solutions, indicating that stearic acid did not react significantly with ZnO. The presence of a novel absorption peak at 1397 cm^−1^ suggests a potential formation of a new chemical bond between stearic acid and ZnO nanoparticles. Comparative analysis of FT-IR spectra from various preparation methods indicates the significant modifying influence of stearic acid on ZnO, suggesting their linkage through covalent bonds rather than mere surface adsorption. This modification leads to the replacement of hydroxyl groups on pure zinc oxide by rigid esters, consequently rendering it hydrophobic.

The analysis of FTIR spectra confirmed that chemical reactions occurred when ZnO was mixed with stearic acid, which caused ZnO nanoparticles to disperse in the solvent more efficiently and reduced the surface energy of the coating. In addition, the reaction degree of unprocessed, magnetically stirred, and ultrasonically dispersed samples increased successively. The main reaction between zinc oxide and stearic acid can be expressed as follows [45]:

HO − ZnO − OH + CH_3_(CH_2_)_16_COOH → CH_3_(CH_2_)_16_COO − ZnO − OOC(CH_2_)_16_ CH_3_ + H_2_O

Compared to magnetic stirring, ultrasonic dispersion could greatly shorten the preparation time and improve the coating performance. Therefore, ultrasonic dispersion was selected as the optimal dispersion method of the coating solution.

#### 3.1.2. Application Method

To determine the optimum application method, the contact angles of the ZnO nanoparticle hydrophobic materials coatings applied by spraying, dipping, and brushing methods were measured. The ultrasonic dispersion (UD) instrument method was used for dispersion.

**Figure 8:**
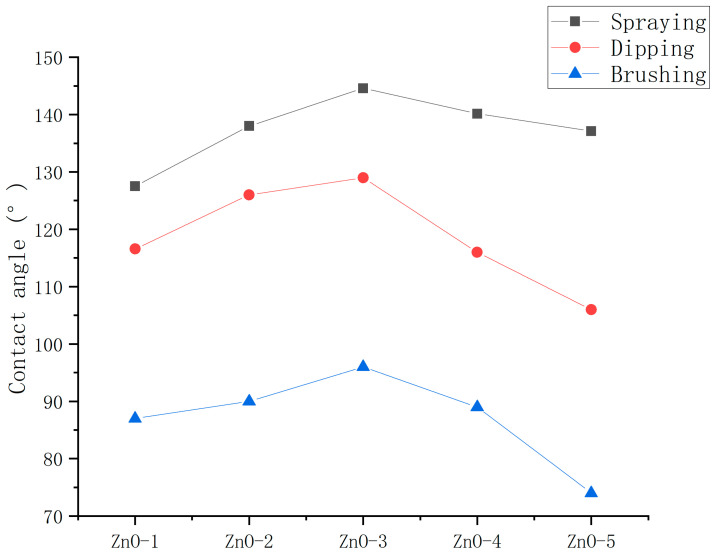
Contact angles of ZnO hydrophobic materials applied by spraying, dipping, and brushing methods.

As shown in Figure 8, the hydrophobicity of coatings prepared by spraying, dipping and brushing methods decreased successively. Moreover, the optimum mass ratios of nanoparticles to stearic acid of the hydrophobic materials applied by spraying, dipping, and brushing methods were the same, indicating that application method did not influence the optimum mass ratios of nanoparticles to stearic acid of the hydrophobic materials.

**Figure 9:**
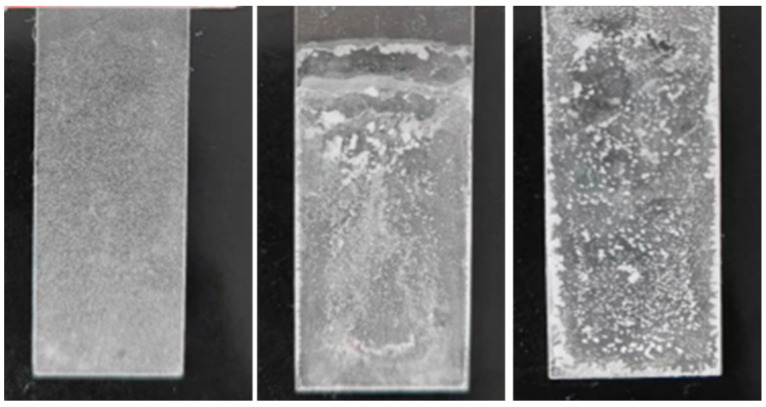
Surface morphology of spraying (**left**), dipping (**middle**), and brushing (**right**) methods.

What needs clarification is the coating testing performed on asphalt concrete, and Figure 9 shows more clearly the difference in coating morphology. As shown in Figure 9, the coating prepared by the spraying method was even, it had a consistent thickness, and its surface was a milky white film. Moreover, the contact angle data at each position had small discreteness, indicating that its hydrophobicity was uniform. The coating prepared using the dipping method was uneven; the nanomaterials were more concentrated in the middle part. Further, some materials agglomerated to form milky white bumps, which was not conducive to the hydrophobicity of the coating. In addition, some areas were not available for contact angle measurement, and the contact angle data at each position had large discreteness. The surface of the coating prepared by the brushing method was uneven and had many milky white protrusions; in particular, the material aggregated more at the edges. Moreover, the overall coating was thick, and most areas were unavailable for contact angle measurement.

From the perspective of engineering practice, only the spraying and brushing methods were applicable. Moreover, the spraying method had better hydrophobicity and was more economical than the brushing method. Therefore, the spraying method was designated as the optimal method to apply the solution.

To sum up, the best method for coating preparation consisted of ultrasonic dispersion and spraying.

#### 3.1.3. Introduction Method for Epoxy Resin

To determine the optimum introduction method for epoxy resin, the contact angles of the ZnO/epoxy hybrid coating and ZnO/epoxy layered coating were measured. The ultrasonic dispersion (UD) instrument method was used for dispersion. The spraying method was used for application.

**Figure 10:**
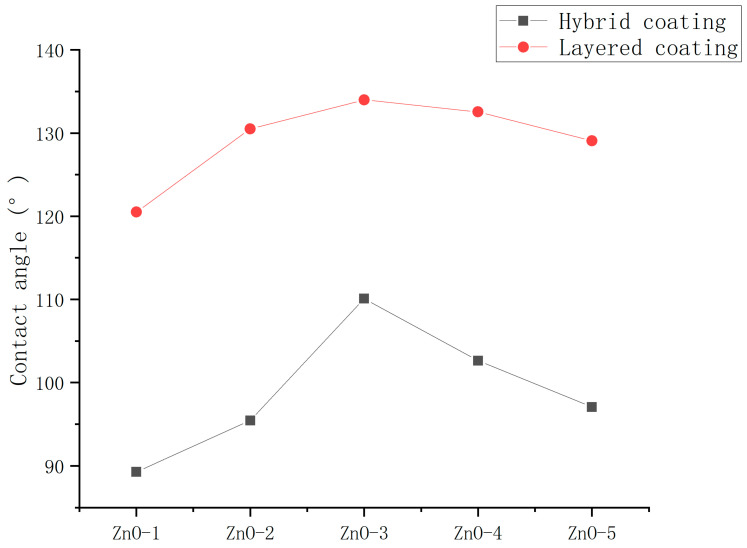
Contact angles of ZnO/epoxy hybrid coating and ZnO/epoxy layered coating.

As shown in Figure 10, the hydrophobicity and durability of the layered coatings were significantly better than those of hybrid coatings. Possibly, on account of the layered arrangement, the epoxy resin did not coat the nanoparticles and the nanoparticles could be exposed to shape the rough surface.

To sum up, the preparation method of nanoparticle/epoxy coating was ultrasonic dispersion and layered spraying.

### 3.2. Contact Angles of Asphalt Concrete with Nanocomposite Coatings

#### 3.2.1. Influence of Mass Ratio of Nanoparticles to Stearic Acid on Contact Angle

**Figure 11:**
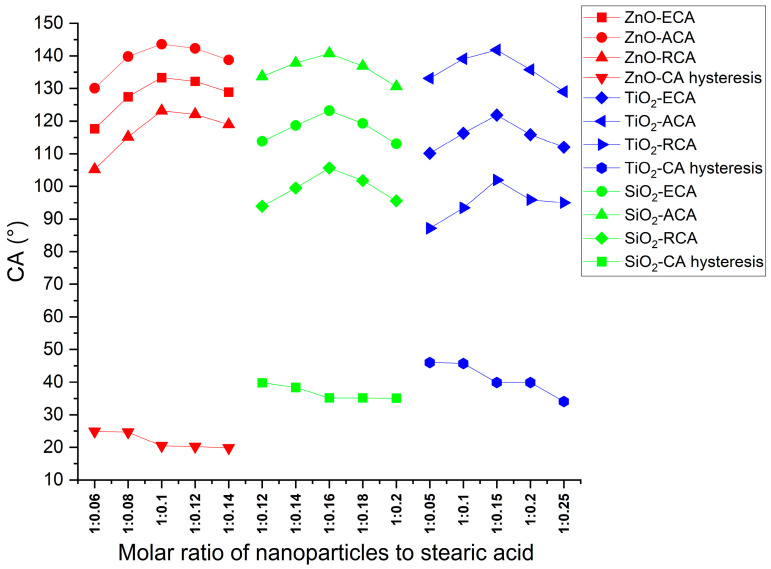
Contact angles of coatings with different molar ratios of nanoparticles to stearic acid.

The contact angles for the samples in Table 2 are shown in Figure 11; the mass ratio of nanoparticles to stearic acid for each sample is listed in Table 2.

Figure 11 shows the contact angles of coatings with different mass ratios of nanoparticles to stearic acid. The mass ratios of stearic acid had a great influence on the ECA of coatings. Whether ZnO, TiO_2_, or SiO_2_ nanoparticles, the ECA increased first and then decreased with the increase in stearic acid. The optimum mass ratios of nanoparticles to stearic acid of the ZnO, TiO_2_, and SiO_2_ coatings were 2.86:1, 1.87:1, and 1.32:1, respectively. This may be because, if the amount of stearic acid was less than the optimum value, the nanoparticles could not react with stearic acid sufficiently. On the other hand, if the amount of stearic acid was more than the optimum value, the hydrophilic carboxyl group in the unreacted stearic acid and the hydrophobic interactions of stearic acid reduced the hydrophilicity of the coating [29]. Among the ZnO, TiO_2_, and SiO_2_ coatings, ZnO exhibits the highest ECA, signifying superior hydrophobicity.

The decrease in CAH across the ZnO, TiO_2_, and SiO_2_ coatings with increasing stearic acid content suggests that stearic acid contributes to enhancing the hydrophobicity. This enhancement can be attributed to the modification of nanoparticles by stearic acid. ZnO coatings display the lowest CAH, followed by SiO_2_ and TiO_2_ coatings, suggesting that ZnO coatings possess the surface roughness most conducive to achieving hydrophobicity.

#### 3.2.2. Influence of Dosage of Nanoparticle to Hydrophobic Materials on Contact Angle

After determining the optimum mass ratios of nanoparticles to stearic acid, we researched the influence of dosage of nanoparticle to hydrophobic materials on contact angle. In all test samples, the mass ratio of nanoparticles to stearic acid was set according to the previously determined optimal ratio. The dosage of nanoparticles to hydrophobic materials refers to the ratio of the mass of the nanoparticle to the mass sum of the nanoparticle, stearic acid, and anhydrous ethanol. The mass of each substance in the hydrophobic materials is shown in Table 2.

**Figure 12:**
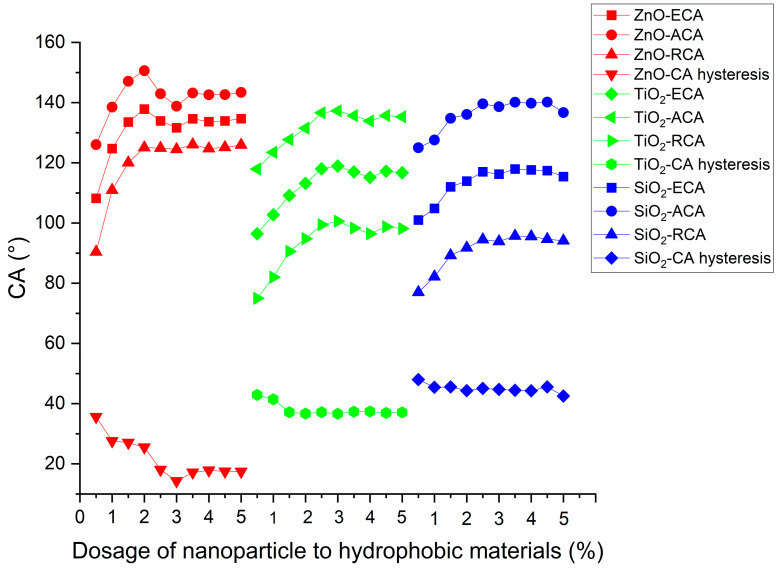
Contact angles of coatings with different dosages of nanoparticle to hydrophobic materials.

Figure 12 shows the contact angles of asphalt concrete with different dosages of nano-ZnO, nano-TiO_2_, and nano-SiO_2_. The contact angle increased first and then saturated with the increase in nanoparticle dosage. It is possible that, as the nanoparticle content increases, the aggregation of nanoparticles becomes more serious, which makes the dispersion of nanoparticles more difficult and the hydrophobicity growth rate of the coating worse [21]. Moreover, when the dosage of ZnO nanoparticles exceeded 3.5%, and for TiO_2_ nanoparticles surpassed 3.5%, as well as SiO_2_ nanoparticles surpassed 3%, the equilibrium contact angle (ECA) exhibited saturation, reaching high values of approximately 132°, 119°, and 117.0°, respectively. In comparison with nano-TiO_2_ and nano-SiO_2_, nano-ZnO was more effective in improving contact angles.

As the nanoparticle content increased, the contact-angle hysteresis (CAH) of ZnO, TiO_2_, and SiO_2_ coatings decreased and eventually reached a plateau at a low value. This trend suggests that nanoparticles contribute positively to enhancing surface roughness and hydrophobicity in the coatings. Notably, the zinc oxide coating exhibits the lowest CAH and the highest equilibrium contact angle (ECA), signifying its superior hydrophobicity compared to the SiO_2_ and TiO_2_ coatings.

### 3.3. Fourier-Transform Infrared (FT-IR) Spectroscopy Analysis

To investigate the effect of different nanomaterials on the hydrophobicity of the coating, the molecular structure of the coatings was analyzed using FTIR. To get rid of impurities, the nanomaterials were centrifuged, and cleaned with distilled water and alcohol.

**Figure 13:**
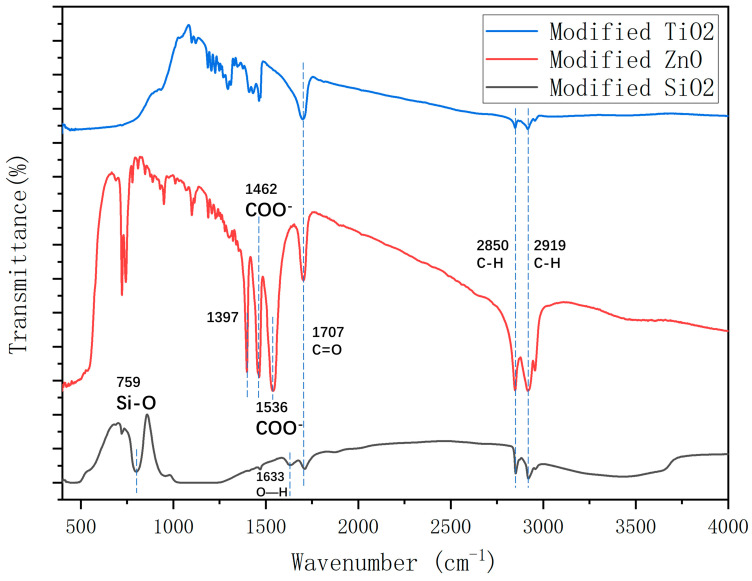
FTIR spectra for modified TiO_2_, ZnO, and SiO_2_.

As shown in Figure 13, the alkyl group in stearic acid was grafted onto the nanoparticle surface because the peaks at 2919 and 2850 cm^−1^ were attributed to the asymmetric stretching vibration absorption of the alkyl group [44]. The peaks at 2919 and 2850 cm^−1^ were assigned to the bending vibration of C–H, which indicates that there were hydroxyl groups and adsorbed water on the surface of SiO_2_ [48]. The presence of the stretching vibration absorption peak of C=O at 1707 cm^−1^, a characteristic peak in stearic acid’s molecular structure, indicates the existence of stearic acid on the surface of the nanoparticles [45].

It was found that the peak areas of TiO_2_ and SiO_2_ were significantly smaller than that of ZnO, indicating that the reaction degree of TiO_2_ and SiO_2_ with stearic acid was significantly lower than that of ZnO. This could explain the lower hydrophobicity of the TiO_2_ and SiO_2_ coatings.

### 3.4. Scanning Electron Microscopy (SEM) Analysis

**Figure 14:**
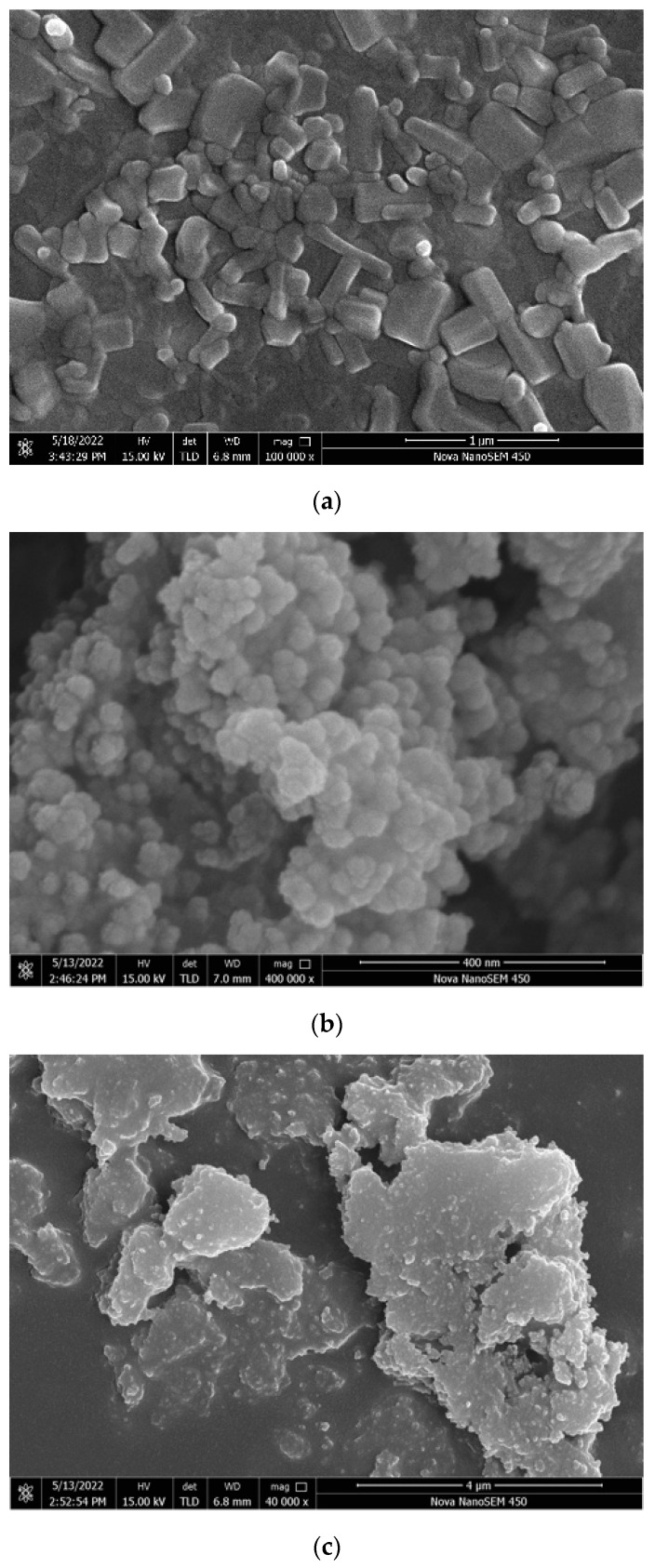

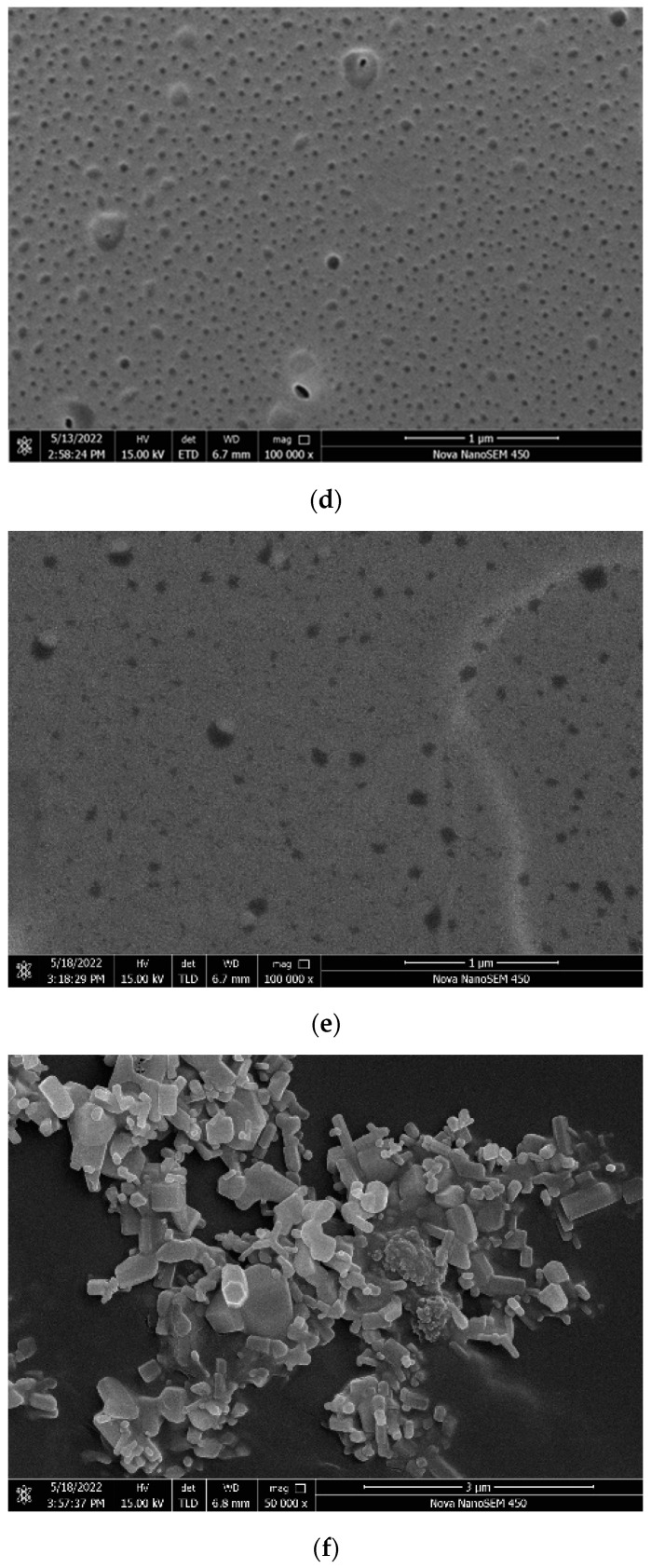
Scanning electron microscopy image: (**a**) ZnO coating; (**b**) SiO_2_ coating; (**c**) TiO_2_ coating; (**d**) epoxy coating; (**e**) ZnO/epoxy hybrid coating; and (**f**) ZnO/epoxy layered coating.

Surface roughness is an important factor affecting the hydrophobicity of the coating. According to the Wenzel model [49], hydrophobicity is related to the effect roughness. As shown in Figure 14, there are serious differences in the roughness of the surface of each coating. The surfaces of the ZnO, SiO_2_, and TiO_2_ coatings are covered with nanoparticles, which are interlaced with each other, and there are enough voids to create conditions for an increase in the contact angle. The surface morphology of the three nanoparticle coatings exhibited significant disparities. The ZnO and SiO_2_ coatings displayed uniform particle distribution with well-defined contours for each nanoparticle. These irregularities, such as indentations and bumps, contributed to a micrometer-scale roughness structure, enhancing hydrophobicity. In contrast, the TiO_2_ nanoparticles formed substantial agglomerations without clear contours, hindering the formation of the rough grooves essential for the Wenzel or Cassie models in the TiO_2_ coatings [50,51]. The superior hydrophobicity of the ZnO coatings and the comparatively lower hydrophobicity of TiO_2_ coatings can be attributed to their respective surface characteristics. The TiO_2_/epoxy hybrid coating and epoxy coating exhibit smooth surfaces akin to the epoxy coating itself. Although minor potholes are present, the overall surface roughness remains insufficient. This suggests that the epoxy resin entirely conceals the surface roughness structure of the nanoparticles, resulting in the loss of hydrophobicity in the coating. The ZnO/epoxy layered coating has a layered structure. According to the SEM image, the white bumps are modified zinc oxide particles and the black smooth part is epoxy resin. The uneven zinc oxide particles improve the hydrophobicity, and the underlying epoxy resin ensures the coating adhesion and abrasion resistance.

### 3.5. Ice Adhesion Strength of Asphalt Concrete with Nanocomposite Coatings

**Figure 15:**
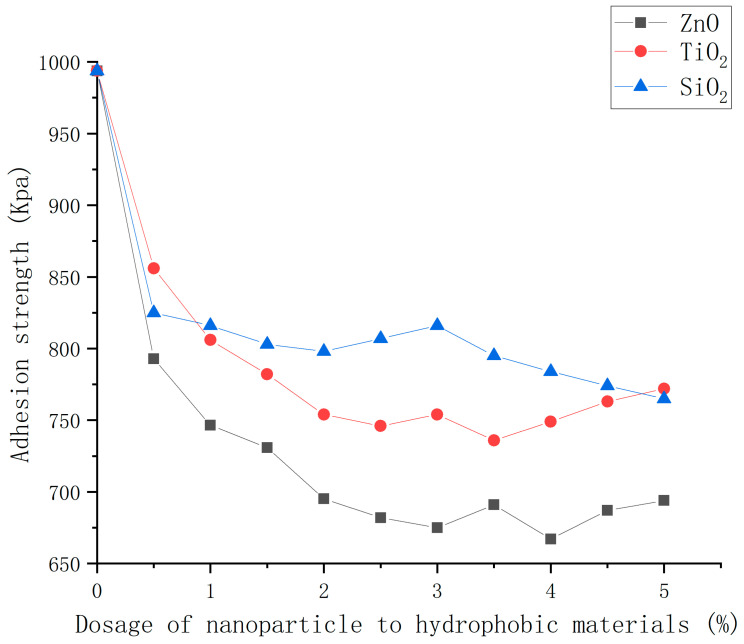
Adhesion strength of coatings with different dosages of nanoparticle to hydrophobic materials.

The adhesion strength between the ice and coating under different nanoparticle dosages was tested. The results are shown in Figure 15.

From Figure 15, it can be seen that, overall, for asphalt concrete with three hydrophobic coatings, the adhesion strength of ice and pavement decreased with the increase in nanoparticle dosage. However, when the dosage of nanoparticles was higher, the adhesion strength was shown to saturate to a low value. Their tendency to aggregate increased, impeding the formation of adequate rough grooves. Consequently, this limitation hampered the nanoparticles’ ability to effectively generate roughness, ultimately diminishing or even negating their anti-icing effect [21]. When the dosages of nanoparticle were 4.0, 3.5, and 5.0 wt% for ZnO, TiO_2_, and SiO_2_, the adhesion strength of three types of coated concrete reached their approximate stable values of 665, 735, and 765 kPa, respectively. The ZnO, TiO_2_, and SiO_2_ coatings helped reduce the adhesion strength by 32.4%, 25.4%, and 22.6% compared with that of ordinary asphalt concrete. The consistent adhesion strength observed in ZnO coatings, being the lowest among the three, followed by TiO_2_ and SiO_2_, indicates that ZnO coatings demonstrate the most robust anti-icing properties, with TiO_2_ and SiO_2_ following in lesser degrees of effectiveness. Overall, the ZnO hydrophobic coating has the best effect of reducing adhesion and moderate dosage.

### 3.6. Icing Proportion of Asphalt Concrete with Nanoparticle Coatings

**Figure 16:**
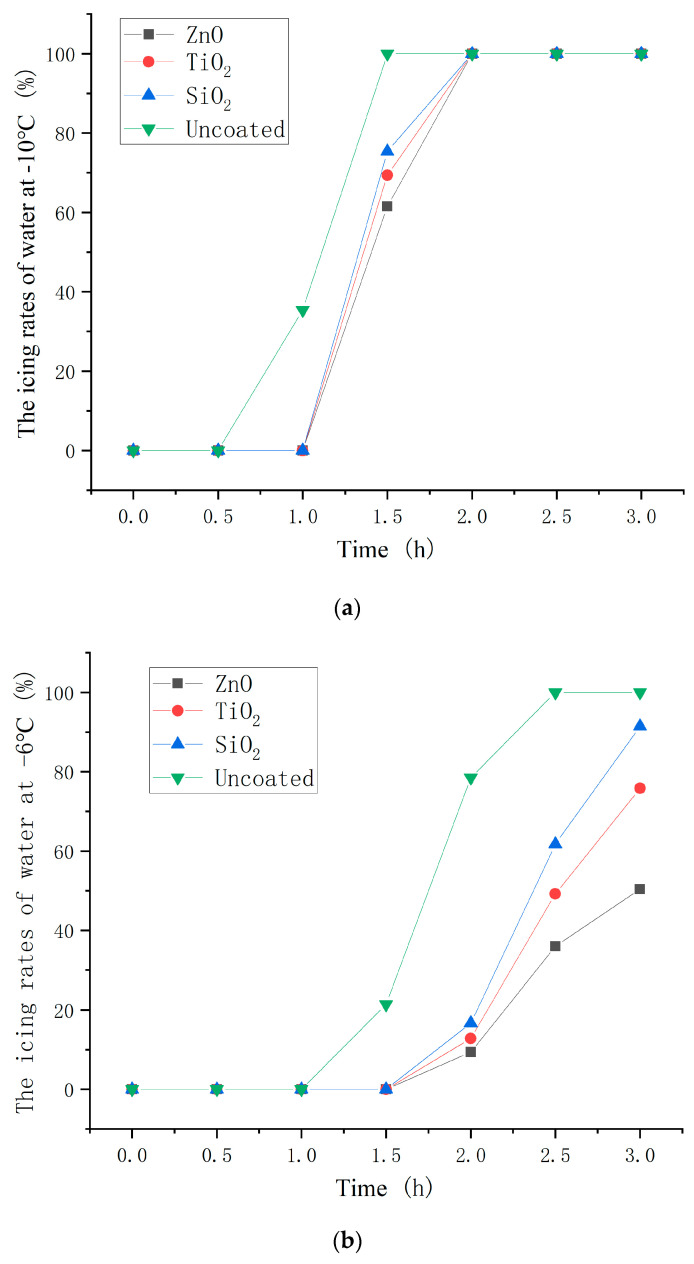

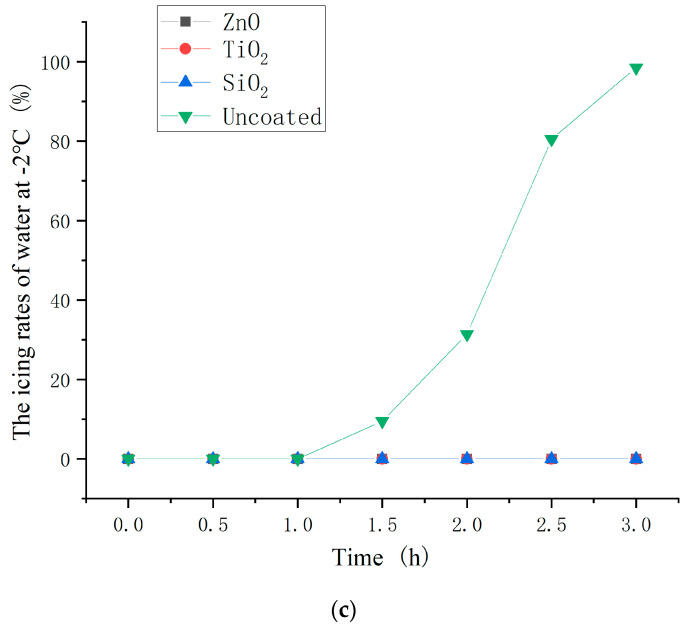
Icing time of water on different asphalt concrete at (**a**) −10 °C; (**b**) −6 °C; and (**c**) −2 °C.

The icing proportion of water on the asphalt concrete with the optimum dosages of nanoparticle to hydrophobic materials (2.0, 3.0, and 4.0 wt% for ZnO, TiO_2_, and SiO_2_ coating) were measured at −10, −6, and −2 °C. The test results are shown in Figure 16. The icing proportion was calculated as the ratio of the mass of ice to the total mass of ice and water.

As shown in Figure 16, the beginning icing time of coated asphalt concrete was significantly later than that of uncoated asphalt concrete and the icing proportion of coated asphalt concrete was less than that of uncoated asphalt concrete at the same point in time. This indicates that the hydrophobic coating has a good inhibiting effect on icing. Moreover, the inhibiting effect on icing of the ZnO, TiO_2_, and SiO_2_ hydrophobic coatings decreased in turn. In the environmental contexts characterized by temperatures of −10 °C and −2 °C, the hydrophobic coating demonstrated a notable extension of the initiation time of ice formation, ranging from a substantial 30 min to a considerable minimum of 2 h, respectively. This signifies the potential of hydrophobic coatings to effectively delay the onset of ice formation, thereby mitigating ice accumulation on road surfaces and, consequently, affording additional time for de-icing procedures.

### 3.7. Contact Angle Attenuation of Coatings with Different Epoxy Resin Contents

We investigated the impact of epoxy resin on the durability test of coatings. All test samples maintained a mass ratio of nanoparticles to stearic acid and nanoparticle dosage consistent with the previously established optimal ratios. Table 3 presents the mass of each component in the hydrophobic materials, Table 4 showing the mass ratio of each substance in relation to various mass ratios of epoxy resin to nanoparticles.

As a comparison, the contact angle of pure epoxy resin cured without nanoparticle deposition was measured to be 31.7°, indicating that epoxy resins were not hydrophobic.

**Figure 17:**
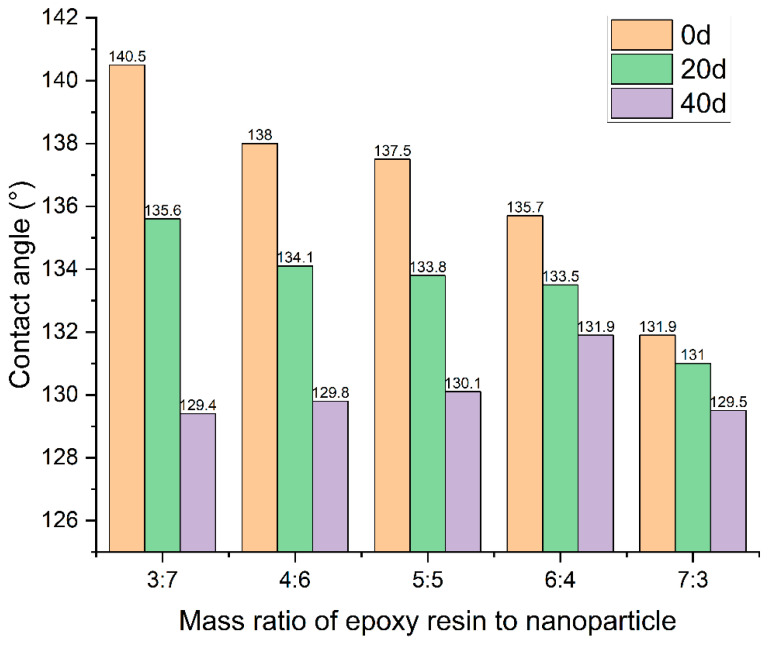
Contact angles of coatings with different mass ratios of epoxy resin to ZnO nanoparticle over time.

The test results of the contact angles of the ZnO coatings with different mass ratios of epoxy resin to nanoparticle over time are shown in Figure 17. It can be seen that the contact angle of the coating was gradually reduced due to the effect of vehicle load and environment, which indicates that the hydrophobicity of the coating was reduced. Moreover, the contact angle decreased with the increased dosage of the mass ratio of epoxy resin to nanoparticle, indicating that the epoxy resin significantly improved the durability of the coating.

Notably, coatings characterized by higher epoxy resin dosages exhibited comparatively smaller contact angles. This phenomenon can be attributed to the saturation of epoxy resin within the interstices between nanoparticles and water droplets, resulting in a reduction in the coating’s overall hydrophobicity. This nuanced relationship underscores the intricate interplay between epoxy resin content and the resultant hydrophobic properties of the coating, thus offering valuable insights into the optimization of coating formulations for enhanced durability.

The contact angle of the hydrophobic coatings remained significantly higher than that of pure epoxy resin even after 40 days. This observation indicates that the nanoparticles retained their hydrophobic properties and continued to contribute to the coatings’ hydrophobicity over time.

## 4. Conclusions

In this study, we investigated the coating preparation, coating hydrophobicity, and anti-icing performance of nanoparticle/epoxy coatings through contact angle test, adhesion test, simulation test of icing, and durability test of coatings. Based on the results and analysis, the following main conclusions can be drawn.

1. The optimum preparation method of nanoparticle/epoxy coating is ultrasonic dispersion and layered spraying. This is because the reaction degree of nanoparticles and stearic acid of unprocessed, magnetically stirred, and ultrasonically dispersed samples increased successively. In addition, layered spraying makes nanoparticles exposed to shape the rough surface.
2. The mass ratio of nanoparticles to stearic acid and dosage of nanoparticle to hydrophobic materials have a significant impact on the contact angle of the hydrophobic coating. If the amount of stearic acid was more than the optimum value, the hydrophilic carboxyl group in the unreacted stearic acid and the hydrophobic interactions of stearic acid reduced the hydrophilicity of the coating. In addition, as the nanoparticle content increases, the aggregation of nano-particles becomes more serious, which makes the dispersion of nanoparticles more difficult and the hydrophobicity growth rate of the coating worse.
3. The FTIR analysis shows that the hydroxyl group on the surface of nanoparticles was replaced by the carboxyl group of stearic acid and it existed on the surface of nanoparticles in the form of coordination adsorption. The reaction degree of TiO_2_ and SiO_2_ with stearic acid was lower than that of ZnO, which could explain the low hydrophobicity of the TiO_2_ and SiO_2_ coatings.
4. The SEM analysis shows that the ZnO/epoxy layered coating has a layered structure. The lower layer of the epoxy resin solution can enhance the durability of the coating and the upper layer of the zinc oxide solution can form a rough hydrophobic surface.
5. The hydrophobic coating can reduce the adhesion strength of ice and asphalt concrete, which decreases with the increase in nanoparticle dosage.
6. The hydrophobic coating has a good inhibiting effect on icing. Moreover, the inhibiting effect on icing of the ZnO, TiO_2_, and SiO_2_ hydrophobic coatings decreases in turn. In the environments of −10 and −2 °C, the hydrophobic coating delayed the beginning icing time by up to 30 min and at least 2 h, respectively.
7. In coating durability tests, the attenuation of contact angle decreased with the increased dosage of the mass ratio of epoxy resin to nanoparticle, indicating that the epoxy resin significantly improved the durability of the coating.

Overall, these results suggest that nanoparticle hydrophobic coatings may be applied to the road surface more easily and with greater durability by using epoxy resin.

## Figures and Tables

**Table 1:** Basic properties of Pen70 asphalt.

Properties	Pen70 Asphalt
Penetration (25 °C)/0.1 mm	66.8
Softening point/°C	49.4
Ductility (15 °C)/cm	145.7

**Table 2:** The mass of each substance in the hydrophobic materials.

Sample		Nanoparticle (g)	Stearic Acid (g)	Mass Ratio of Nanoparticle to Stearic Acid	Molar Ratio of Nanoparticle to Stearic Acid	Anhydrous Ethanol (mL)	Epoxy Resin (g)
ZnO-1	ZnO (g)	5	1.05	4.76:1	1:0.06	100	5
ZnO-2		5	1.40	3.57:1	1:0.08	100	5
ZnO-3		5	1.75	2.86:1	1:0.1	100	5
ZnO-4		5	2.10	2.38:1	1:0.12	100	5
ZnO-5		5	2.45	2.04:1	1:0.14	100	5
SiO_2_-1	SiO_2_ (g)	5	2.84	1.76:1	1:0.12	100	5
SiO_2_-2		5	3.32	1.51:1	1:0.14	100	5
SiO_2_-3		5	3.79	1.32:1	1:0.16	100	5
SiO_2_-4		5	4.27	1.17:1	1:0.18	100	5
SiO_2_-5		5	4.74	1.05:1	1:0.2	100	5
TiO_2_-1	TiO_2_ (g)	5	0.89	5.62:1	1:0.05	100	5
TiO_2_-2		5	1.78	2.81:1	1:0.1	100	5
TiO_2_-3		5	2.67	1.87:1	1:0.15	100	5
TiO_2_-4		5	3.56	1.40:1	1:0.2	100	5
TiO_2_-5		5	4.45	1.12:1	1:0.25	100	5

**Table 3:** The mass ratio of each substance in the hydrophobic materials with different dosages of nanoparticle to hydrophobic materials.

Dosage of Nanoparticle to Hydrophobic Materials (%)		Nanoparticle (g)	Stearic Acid (g)	Anhydrous Ethanol (g)	Epoxy Resin (g)
0.50	ZnO(g)	5	1.75	993.25	5
1.00		5	1.75	493.25	5
1.50		5	1.75	326.58	5
2.00		5	1.75	243.25	5
2.50		5	1.75	193.25	5
3.00		5	1.75	159.92	5
3.50		5	1.75	136.11	5
4.00		5	1.75	118.25	5
4.50		5	1.75	104.36	5
5.00		5	1.75	93.25	5
0.50	SiO_2_(g)	5	3.79	991.21	5
1.00		5	3.79	491.21	5
1.50		5	3.79	324.54	5
2.00		5	3.79	241.21	5
2.50		5	3.79	191.21	5
3.00		5	3.79	157.88	5
3.50		5	3.79	134.07	5
4.00		5	3.79	116.21	5
4.50		5	3.79	102.32	5
5.00		5	3.79	91.21	5
0.50	TiO_2_(g)	5	2.67	992.33	5
1.00		5	2.67	492.33	5
1.50		5	2.67	325.66	5
2.00		5	2.67	242.33	5
2.50		5	2.67	192.33	5
3.00		5	2.67	159.00	5
3.50		5	2.67	135.19	5
4.00		5	2.67	117.33	5
4.50		5	2.67	103.44	5
5.00		5	2.67	92.33	5

**Table 4:** The mass ratio of each substance in the hydrophobic materials with different mass ratios of epoxy resin to nanoparticle.

Mass Ratios of Epoxy Resin to Nanoparticle		Nanoparticle (g)	Stearic Acid (g)	Anhydrous Ethanol (g)	Epoxy Resin (g)
7:3	ZnO (g)	5	1.75	243.25	2.14
6:4		5	1.75	243.25	3.33
5:5		5	1.75	243.25	5.00
4:6		5	1.75	243.25	7.50
3:7		5	1.75	243.25	11.67

## Data Availability

Data are contained within the article.
